# Supplementary material for: Heat adaptation of phage T7 under an extended genetic code
Source: Virus Evol. 2021 Dec 1;7(2):veab100. doi: 10.1093/ve/veab100 (PMC8923235; doi:10.1093/ve/veab100)
Supplement: veab100_Supp [file veab100_supp.zip › Sequencing_Analysis.zip!/Sequencing_Analysis/A1/evidence/RA_19.html]

BRESEQ :: Evidence


| Read alignment evidence... | | | | | | | | | | | |
| --- | --- | --- | --- | --- | --- | --- | --- | --- | --- | --- | --- |
|  | seq id | position | | ref | new | freq | score (cons/poly) | reads | annotation | genes | product |
| \* | NC\_001604.1 | 13,582 | 0 | T | A | 20.7% | 3838.1 / inf | 2343 | intergenic (–/–) | *–/–* | –/– |
| Reads supporting (aligned to +/- strand):  **ref** base T (885/953);  **new** base A (218/263);  **total** (1110/1233) | | | | | | | | | | | |
| Fisher's exact test for biased strand distribution *p*-value = 2.82e-01 | | | | | | | | | | | |
| Kolmogorov-Smirnov test that lower quality scores support variant *p*-value = 4.08e-02 | | | | | | | | | | | |

Only 100 of 2383 total aligned reads displayed.  

`TTCCAGACGACTACGAGAAGTTTCGCTGGAGAGTCCCATTCTAATACGACTCACTAAAGGAGACACACCATGTTCAAACTGATTAAGAAGTTAGGCCAACTGCTGGTTCGTATGTACAACGTGGAAGCCAAGCGACTGAACGATGAGGCTCGTAAAGAGGCCACACAGTCACGCGCTCTGGCGATTCGCTCCAACGAACTGGCTGACAGTGCATCCACTAAAGTTACCGAGGCTGCCCGTGTGGCAAACCAAGCTCAACAGCTTTCCAAATTCTTTGAGTAATCAAACA‑GGAGAAACCATTATGTCTAACGTAGCTGAAACTATCCGTCTATCCGATACAGCTGACCAGTGGAACCGTCGAGTCCACATCAACGTTCGCAACGGTAAGGCGACTATGGTTTACCGCTGGAAGGACTCTAAGTCCTCTAAGAATCACACTCAGCGTATGACGTTGACAGATGAGCAAGCACTGCGTCTGGTCAATGCGCTTACCAAAGCTGCCGTGACAGCAATTCATGAAGCTGGTCGCGTCAATGAAGCTATGGCTATCCTCGACAAGATTGATAACTAAGAGTGGTATC  >  NC_001604.1/13283‑13863`  

                                                                                                                                                                                                                                                                                                            `|`                                                                                                                                                                                                                                                                                           
`ttCCAGACGACTACGAGAAGTTTCGCTGGAGAGTCCCATTCTAATACGACTCACTAAAGGAGACACACCATGTTCAAACTGATTAAGAAGTTAGGCCAACTGCTGGTTCGTATGTACAACGTGGAAGCCAAGCGACTGAACGATGAGGCTCGTAAAGAGGCCACACAGTCACGCGCTCTGGCGATTCGCTCCAACGAACTGGCTGACAGTGCATCCACTAAAGTTACCGAGGCTGCCCGTGTGGCAACCCAATCTCAACAACTTTCCAACTTCTTTTAGTAATCAAACA‑AGCAAAACCATT                                                                                                                                                                                                                                                                                          >  1:253710‑M1/1‑301 (MQ=255)`  
     `aacgacaACGAGAAGTTTCGCTGGAGAGTCACATTCTAATACGACTCACTAAAGGAGACACACCATGTTCAAACAGATTAAGAAATTAGGCCAACTGCTGGTTCGTATGTACAACGTGGAAGCCAAGCGACTGAACAATGAGGCTCGTAAAGAGGCCACACAGTCACGCGCTCTGGCGATTCGCTCCAACGAACTGGCTGACAGTGCATCCACTAAAGTTACCGAGGCTGCCCGTGTGGCAAACCAAGCTCAACAGCTTTCCAAATTCTTTGAGTAATCAAACA‑GGAGAAACCATTATGat                                                                                                                                                                                                                                                                                     <  1:85524‑M1/300‑3 (MQ=255)`  
                   `gTTTCGCTGGCGAGTCCCATTCTAATACGACTCAATAAAGTAGACACACCATGTTCAAACTGATTAAGAAGTTAGGCCAACTGCTGGTTCGTATGTACAACGTGGAACCCAAGCGACTGAACGATGAGGCTCGTAAAGAGGCCACACAGTCACGCGATCTGCAGATTCGCTCCAACGAACGAGCAGAAAGTGACTCCTCTAAAGTTACAGAGGCCACACGGGTGGCAACACAAGCTCAAAAGCTCTCCCAATTCTTTGAGTCATCCACCA‑GGAGAACCCCTTCTgcacgctgtctcatata                                                                                                                                                                                                                                                                       >  1:498389/1‑285 (MQ=255)`  
                   `gTTGCGCTGGAGATTCCCAGTCTAAGACGACTCACTAAAGGAGACACACCATGTTCAAACTGATTAAGAAGTTAGGCCAACTGCTGGTTCGTATGTACAACGTGGAAACCAAGCGACTGAACGATGAGGCTCGGAAAGAGGCCACACAGTCACGCTCTCTGGCGATTCGCTCTAACGAACTGGCTGACAGTGCATCCACTAAAGTTAACGAGGCTGCCCGTGTGGAAAACCAAGCTCAACAGCTTTCCAAATTCTTTGAGTAATCAAACA‑TGAGAAACCATTAGGatcgtttctgacatcg                                                                                                                                                                                                                                                                       <  1:208230‑M1/301‑17 (MQ=255)`  
                      `tCGCTGGAGAGTCCCATTCTAATACGACTCACTAAAGGAGACACACCATGTTCAACCTGCTTAAGAAGTTAGGCCAACTGCTGGTTCGTATGTACAACGTGGAAGCCAAGCGACTGAACGATGAGGCTCGTAAAGAGGCCACACAGTCACGCGCTCTGGCGATTCGCTCCAACGAACTGGCTGACAGTGCATCCACTAACGTTACCGAGGCTGCCCGTGTGGCAAACCAAGCTCAACAGCTTTCCAAATTCTTTGAGTAATCAAACA‑GGAGAAACCATTATGatcgtttctgacatcgaaa                                                                                                                                                                                                                                                                    >  1:56957‑M1/1‑282 (MQ=255)`  
                      `tCGCTGGAGAGTCCCATTCTAATACGACTCACTAAAGGAGACACACCATGTTCAAACTGATTAAGAAGTTAGGCCAACTGCTGGTTCGTATGTACAACGTGGAAGCCAAGCGACTGAACGATGAGGCTCGTAAAGAGGCCACACAGTCACGCGCTCTGGCGATTCGCTCCAACGAACTGGCTGACAGTGCATCCACTAAAGTTACCGAGGCTGCCCGTGTGGCAAACCAAGCTCAACAGCTTTCCAAATTCTTTGAGTAATCAAACA‑GGAGAAACCATTATGatcgtttctgacatcgaag                                                                                                                                                                                                                                                                    >  1:126417‑M1/1‑282 (MQ=255)`  
                           `ggAGAGTCCCATTCTAATACGACTCACTAAAGGAGACACACCATGTTCAAACTGATTAAGAAGTTAGGCCAACTGCTGGTTCGTATGTACAACGTGGAAGCCAAGCGACTGAACGATGAGGCTCGTAAAGAGGCCACACAGTCACGCGCTCTGGCGATTCGCTCCAACGAACTGGCTGACCGTGCATCCACTAAAGTTACCGAGGCTGCCCGTGTGGCAAACCAAGCTCAACCGCTTTCCAAATTCTTTGATTAATCAAACA‑GGAGAAACCATTATGatcgtttctgactgtctcttatac                                                                                                                                                                                                                                                               >  1:211355‑M1/1‑277 (MQ=255)`  
                                `gTCCCATTCTAATACGACTCACTAAAGGAGACACACCATGTTCAAACTGATTAAGAAGATAGGCCAACTGCTGGTACGTAAGTACAACGTGGAAGCCAAGCGACTGAACGAGGAGGCTCGTAAAGAGGCCACACAGTCACGCGCTCTGGCGATTCGCTCCAACGAACTGGCTGACAGTGCATCCACTAAAGTTACCGAGGCTGCACGTGAGGCAAACCAAGCTCAACAGCTTTCCAAATTCTTTGAGTAATCAAACA‑GGAGAAATCAATAAGatcgattcctgttgcgtataaacagatac                                                                                                                                                                                                                                                          >  1:3682‑M1/1‑272 (MQ=255)`  
                                     `aTTCTAATACGACTCACTAAAGGAGACACACCATGTTCAAACTGATTAAGAAGTTAGGCCAACTGCTGGTTCGTATGTACAACGTGGAAGCCAAGCGACTGAACGATGAGGCTCGTAAAGAGGCCACACAGTCACGCGCTCTGGCGATTTGCTCCAACGAAATGGCTGACAGTGCATCCACTAAAGTTACCCAGGCTGCCCGTGTGGCAAACCAAGCTCAACAGCTTTCCAAATTCTTTGAGTAATCAAACA‑GGAGAAACCATCATGatagtttctgaaatcgaagctaacgccctcttag                                                                                                                                                                                                                                                     >  1:16130‑M1/1‑267 (MQ=255)`  
                                      `ttCTAATACGACTCACTAAAGGAGACACGGCATGATCAAACTGATTAAGAAGTTCGGCCAACTGCTGGTTCGTATTTACAACGTGGAAGCCAGGCGAATGAACGATGATGCTCGTAAAGAGGCCACACAGTCACGCTATCTGGCGACTCGCCCCAACGAACTGGCTGACAGTTCATCCACTAAAGTTACCGAGGCTGTCAGTGTGGCAACCCAAGCTCAACAGCTTTCCAAAGTCTTTGAGTAATCAAACA‑GGAGAAACCATTATGaactattctgacctctaaggtcacgccctcttcac                                                                                                                                                                                                                                                    >  1:80518‑M1/1‑266 (MQ=255)`  
                                      `ttCTAATACGACTCACTAAAGGAGACACACCATGTTCAAACTGATTAAGAAGTTAGGCCAACTGCTGGTTCGTATGTACAACGTGGAAGCCAAGCGACTGAACGATGAGGCTCGTAAAGAGGCCACACAGTCACGCGCTCTGGCGATTCGCTCCAACGAACTGGCTGACAGTGCATCCACTAAAGTTACCGAGGCTGCCCGTGTGGCAAACCAAGCTCAACAGCTTTCCAAATTCTTTGAGTAAT‑ACACACGGAGAAACCATTATGttcgttcatgacatcgaagcctgtcacttatacac                                                                                                                                                                                                                                                    >  1:174629‑M1/1‑266 (MQ=255)`  
                                      `ttCTAATACGACTCACTAAAGGAGACACACCATGTTCAAACTGATTAAGAAGTTAGGCCAACTGCTGGTTCGTATGTACAACGTGGAAGACAAGCGACTGAACGATGAGGCTCGTAAAGAGGCCACACAGTCACGCGCTCTGGCGATTCGCTCCAACGAACTGGCTGACAGTGCATCCACTAAAGTTACCGAGGCTGCCCGTGTGGCCAACCAAGCTCAACAGCTTTCCAAATTCTTTGAGTAATCAAACA‑GGAGAAACCATTATGatcgtttctgacatcgaagctaacgccctcttaga                                                                                                                                                                                                                                                    >  1:62562‑M1/1‑266 (MQ=255)`  
                                       `aagaatacGACTCACTAAAGGAGACAACCCATGTTCAACCTGATTAAGAAGTTAGGCCAACTGCTGGTTCGTAAGTACAACTGAGACGCCTAGCGACTTAACGATTAGGCTCGTAAAGAGGCCACACAGTCACGCGCTCTGGCGATTAGCTCCAACGAACTGGCTGACAGTGCATCCACTAAAGTTACCGAGGCTGCCCGTGGGGCAAACCAAGCTCAACAGCTTTCCAAATTCTTTGAGTAATCAAACA‑GGAGAAACCATTATGatcgtttctgacatcgaagctaacgccctcttagag                                                                                                                                                                                                                                                   <  1:447605‑M1/298‑37 (MQ=255)`  
                                         `tAATACGACTCACTAAAGGAGACACACCATGTTCAAACTGATTAAGAAGTTAGGCCAACTGCTGGTTCGTATGTACAACGTGGAAGCCAAGCGACTGAACGATGAGGCTCGTAAAGAGGCCACACAGTCACGCGCTCTGGCGATTCGCTCCAACGAACTGGCTGACAGTGCATCCACTAAAGTTACCGAGGCTGCCCGTGTGGCAAACCAAGCTCAACAGCTTTCCAAATTCTTTGAGTAATCAAACA‑TGAGAACCCATTATGatcgtttctgacatcgaagctaacgccctcttagagag                                                                                                                                                                                                                                                 >  1:233735‑M1/1‑263 (MQ=255)`  
                                         `tAATACGACTCACTAAAGGAGACACACCATGTTCAAACTGATTAAGAAGTTAGGCCAACTGCTGGTTCGTATGTACAACGTGGAAGCCAAGCGACTGAACGATGAGGCTCGTAAAGAGGCCACACAGTCACGCGCTCTGGCGATTCGCTCCAACGAACTGGCTGACAGTGCATCCACTAAAGTTACCGAGGCTGCCCGTGTGGCAAACCAAGCTCAACAGCTTTCCAAATTCTTTGAGTAATCAAACA‑GGAGAAACCATTATGatcgtttctgacatcgaagctaacgccctcttcgagct                                                                                                                                                                                                                                                 >  1:342411‑M1/1‑263 (MQ=255)`  
                                         `aaatACGACTCACTAAAGGAGACAAACCATGTTCAAACTGATGAAGAAGATAGGCCAACTGATGGTTCGTATGTACAACGTGGAAGCCAAGCGACTGAACTATGAGGCTCGTAAAGAGGCCACACAGTCACGCGCTCTGGCGATTCGCTCCAACGAACTGGCTGACAGTGCATCCACTAAAGTTACCGAGGCTGCCCGTGTGGCAAACCAAGCTCAACAGCTTTCCAAATTCTTTGAGTAATCAAACA‑GGAGAAACCATTATGatcgtttctgacatcgaagctaacgccctcttagagag                                                                                                                                                                                                                                                 <  1:296992‑M1/300‑39 (MQ=255)`  
                                             `aCGACTCACTAAAGGAGACACACCATGTTCAAACTGATTAAGAAGTTAGGCCAACTGCTGGTTCGTATGTACAACGTGGAAGCCAAGCGACTGAACGATGAGGCTCGTAAAGAGGCCACACAGTCACGCGCTCTGGCGATTCGCTCCAACGAACTGGCTGACAGTGCATCCACTAAAGTTACCGAGGCTGCCCGTGTGGCAAACCAAGCTCAACAGCTTTCCAAATTCTTTGAGTAATCAAACA‑GGAGAAACCATTATGatcgtttctgacatcgaagctaacgccctcttagagagcgtc                                                                                                                                                                                                                                             <  1:381839‑M1/301‑43 (MQ=255)`  
                                               `acagcactaAAGGAGACACACCATGTTCAAACTGATTAAGAAGTTAGGCCAACTGCTGGTTCGTATGTACAACGTGGAAGCCAAGCGACTGAACGATGAGGCTCGTAAAGAGGCCACACAGTCACGCGCTCTGGCGATTCGCTCCAACGAACTGGCTGACAGTGCATCCACTAAAGTTACCGAGGCTGCCCGTGTGGCAAACCAAGCTCAACAGCTTTCCAAATTCTTTGAGTAATCAAACA‑GGAGAAACCATTATGatcgtttctgacatcgaagccaacgccctcttagagagcgtcac                                                                                                                                                                                                                                           <  1:289038‑M1/297‑45 (MQ=255)`  
                                                `aCTCACTAAAGGAGACACACCATGTTCAAACTGATTAAGAAGTTAGGCCAACTGCTGGTTCGTATGTACAACGTGGAAGCCAAGCGACTGAACGATGAGGCTCGTAAAGAGGCCACACAGTCACGCGCTCTGGCGATTCGCTCCAACGAACTGGCTGACAGTGCATCCACTAACGTTACCGAGGCTGCCCGTGTGGCAAACCAAGCTCAACAGCTTTCCAAATTCTTTGAGTAATCAAACA‑GGAGAAACCACTATGaacgtttctgaactcgaagctaacgctctcttagagagcgtccat                                                                                                                                                                                                                                          >  1:269097‑M1/1‑256 (MQ=255)`  
                                                   `cACTAAAGGAGACACACCATGTTCAAACTGATTAAGAAGTTAGGCCAACTGCTGGTTCGTATGTACAACGTGGAAGCCAAGCGACTGAACGATGAGGCTCGTAAAGAGGCCACACAGTCACGCGCTCTGGCGATTCGCTCCAACGAACTGGCTGACAGTGCATCCACTAAAGTTGCCGAGGCTGCCCGTGTGGCAAACCAAGCTCAACAGCTTTCCAAATTCTTTGAGTAATCAAACA‑GGAGACACCATTATGatcgtttctgacatcgaagctaacgccctcttagagagcgtcactaag                                                                                                                                                                                                                                       >  1:469371‑M1/1‑253 (MQ=255)`  
                                                   `cACTAAAGGAGACACACCATGTTCAAACTGATTAAGAAGTTAGGCCAACTGCTGGTTCGTATGTACAACGTGGAAGCCAAGCGACTGAACGATGAGGCTCGTAAAGAGGCCACACAGTCACGCGCTCTGGCGATTCGCTCCAACGAACTGGCTGACAGTGCATCCACTAAAGTTACCGAGGCTGCCCGTGTGGCAAACCAAGCTCAACAGCTTTCCAAATTCTTTGAGTAATCAAACA‑GGAGAAACCATTATGatcgtttctgacatcgaagctaacgccctcttagagctgtctcttata                                                                                                                                                                                                                                       >  1:167779‑M1/1‑253 (MQ=255)`  
                                                    `aagaaaggaGACACAACATGTTAAAACTGATTAAGAAGTTAGGCCAACTGCTGGTTCGTATGTACAACGTGGAAGCCAAGCGACTGAACGATGAGGCTCGTAAAGAGGCCACACAGTCACGCGCTCTGGCGATTCGCTCCAACGAACTGGCTGACAGTGCATCCACTAAAGTTACCGAGGCTGCCCGTGTGGCAAACCAAGCTCAACAGCTTTCCAAATTCTTTGAGTAATCAAACA‑GGAGAAACCATTATGatcgtttctgacatcgaagctaacgccctcttagagagcgtcactaagt                                                                                                                                                                                                                                      <  1:386813‑M1/298‑50 (MQ=255)`  
                                                     `ttatcagagacagaccATGTTCAAACTGATTAAGAAGTTAGGCCAACTGCGGGTTCTTATGTACAACGTGGAAGTCAAGCGACTGAACGATGAGACTCGTAAAGAGGCCACACAGTCACGCGCTCTGGCGATTCGCTCCAACGAACTGGCTGACAGTGCATCCACTAAAGTTACCGAGGCTGCCCGTGTGGCAAACCAAGCTCAACAGCTTTCCAAATTCTTTGAGTAATCAAACA‑GGAGAAACCATTATGatcgtttctgacatcgaagctaacgccctcttagagagcgtcactaagtt                                                                                                                                                                                                                                     <  1:284834‑M1/295‑51 (MQ=255)`  
                                                         `aGGAGACACACCATGTTCAAACTGATTAAGAAGTTAGGCCAACTGCTGGTTCGTATGTACAACGTGGAAGCCAAGCGACTGAACGATGAGGCTCGTAAAGAGGCCACACAGTCACGCGCTCTGGCGATTCGCTCCAACGAACTGGCTGACAGTGCATACACTAAAGTTACCGAGGCTGCCAGTGTGGCAAACCAAGCTCAACAGCTTTCCAAATTCTTTGAGTAATCAAACA‑GGAGAAACCATTATGatcgtttctgacatcgaagctaacgcactcttagagagcgtcactaagttccac                                                                                                                                                                                                                                 <  1:452776‑M1/301‑55 (MQ=255)`  
                                                          `tcggagatgtgtataagagacagattaagaagttaggccaactgcTGGTTCGTATGTACAACGTGGAAGCCAAGCGACTGAACGATGAGGCTCGTAAAGAGGCCACACAGTCACGCGCTCTGGCGATTCGCTCCAACGAACTGGCTGACAGTGCATCCACTAAAGTTACCGAGTCTGCACGTGT‑GCAAACCAAGCTCAACAGCTTTCCAAATTCTTTGAGTAATCAAACA‑GGAGAAATCAATATGatcgtttctgacatcgaagctaacgacctcttagagagcgtcactaagttccactg                                                                                                                                                                                                                               <  1:11327‑M1/279‑57 (MQ=255)`  
                                                           `gagaCACACCATGTTCAAACTGATTAAGAAGTTAGGCCAACTGCTGGTTCGTATGTACAACGTGGAAGCCAAGCGACTGAACGATGAGGCTCGTAAAGAGGCCACACAGTCACGCGCTCTGGCGATTCGCTCCAACGAACTGGCTGACAGTGCATCCACTAAAGTTACCGAGGCTGCCCGTGTGGCAAACCAAGCTCAACAGCTTTCCAAATTCTTTGAGTAATCAAACA‑GGAGAAACCATTATGatcgtttctgacatcgaagctaacgccctcttagagagcgtcactaagttccactg                                                                                                                                                                                                                               <  1:408727‑M1/301‑57 (MQ=255)`  
                                                                 `cacCATGTTCAAACTGATTAAGAAGTTAGGCCAACTGCTGGTTCGTATGTACAACGTGGAAGCCAAGCGACTGAACGATGAGGCTCGTAAAGAGGCCACACAGTCACGCGCTCTGGCGATTCGCTCCAACGAACTGGCTGACAGTGCATCCACTAAAGTTACCGAGGCTGCCCGTGTGGCAAACCAAGCTCAACAGCTTTCCAAATTCTTTGAGTAATCAAACA‑GGAGAAACCATTATGatcgtttctgacatcgaagctaacgccctcttagagagcgtaactaagttccactgcggggt                                                                                                                                                                                                                         >  1:41364‑M1/1‑239 (MQ=255)`  
                                                                     `aTGTTCAAACTGATTAAGAAGTTAGGCCAACTGCTGGTTCGTATGTACAACGTGGAAGCCAAGCGACTGAACGATGAGGCTCGTAAAGAGGCCACACAGTCACGCGCTCTGGCGATTCGCTCCAACGAACTGGCTGACAGTGCATCCACTAAAGTTACCGAGGCTGCCCGTGTGGCAAACCAAGCTCCACAGCTTTCCAAATTCTTTGAGTAATCAAACA‑GGAGCAATCAATATGatcgtttcttacatcgaagctaacgccctcttagagagcgccactaagttccactgcggggttatt                                                                                                                                                                                                                     >  1:479137‑M1/1‑235 (MQ=255)`  
                                                                      `tGTTTAAACTGATTAAGAAGTTAGGCCAACTGCTGGTTCGTATGTACAACGTGGAAGCCAAGCGACTGAACGATGAGGCTCGTAAAGAGGCCACACAGTCACGCGCTCTGGCGATTCGCTCCAACGAACTGGCTGACAGTGCATCCACTAAAGTTACCGAGGCTGCCCGTGTGGCAAACCAAGCTCAACAGCTTTCCAAATTCTTTGAGTAATCGAACA‑GGAGAAACCATTATGatcgtttctgacatagaagctaacgccctcttagaaagcgtcactcagttccactgcggggttatct                                                                                                                                                                                                                    >  1:32687‑M1/1‑234 (MQ=255)`  
                                                                      `tGTTCAAACTGATTAAGAAGTTAGGCCAACTGCTGGTTCGTATGTACAACGTGGAAGCCAAGCGACTGAACGATGAGGCTCGTAAAGAGGCCACACAGTGACGCGCTCTGGCGATTCGATCCAACGAACTGGCTGACAGTGCATCCACTAAAGTTACCGAGGCTGCCCGTGTGGCAAACCAAGCTCAACAGCTTTCCAAATTCTTTGAGTAATCAAACA‑GGAGAAATCAATATGatcgtttctgacatcgaagctaacgccctcttcgcgagcgtcactaagttccactgcggggttagct                                                                                                                                                                                                                    >  1:42329‑M1/1‑234 (MQ=255)`  
                                                                      `tGTTCAAACTGATTAAGAAGTTAGGCCAACTGCTGGTTCGTATGTACAACGTGGAAGCCAAGCGACTGAACGATGAGGCTCGTAAAGAGGCCACACAGTCACGCGCTCTGGCGATTCGCTCCAACGAACTGGCTGACAGTGCATCCACTAAAGTTACCGAGGCTGCCCGTGTGGCAAACCAAGCTCAACAGCTTTCCAAATTCTTTTAGTAATCAAACA‑GGAGAAATCAATATGatcgtttctgacatcgaagctaacgcactcttagagagcgtaactaagttccacctgtctcttataa                                                                                                                                                                                                                    >  1:204279‑M1/1‑234 (MQ=255)`  
                                                                       `gTTCAAACTGATTAAGAAGTTAGGCCAACTGCTGGTTCGTATGTACAACGTGGAAGCCAAGCGACTGAACGATGAGGCTCGTAAAGAGGCCACACAGTCACGCGCTCTGGCGATTCGCTCCAACGAACTGGCTGACAGTGCATCCACTAAAGTTACCGAGGCTGCCCGTGTGGCAAACCAAGCTCAACAGCTTTCCAAATTCTTTGAGTAATCAAACC‑GGAGAAACCATTATGatcgtttctgacatcgaagctaacgccctcttagagagcgtcactaagttccactgcggggtctgtct                                                                                                                                                                                                                   >  1:109691‑M1/1‑233 (MQ=255)`  
                                                                        `ttCAAACTGATTAGGAAGTTAGGCCAACTGCTGGTTCGTATGTACAACGTGGAAGCCAAGCGACTGAACGATGAGTCTCGTAAAGAGGCCACACAGTCACGCGCTCTGGCGATTCGCTCCAACGAACTGGCTGACAGTGCATCCACTAAAGTTACCGAGGCTGCCCGTGTGGCAAACCAAGCTCAACAGCTTTCCAAATTCTTTGAGTAATCAAACA‑GGAGAAACCATTATGatagtttctgacatcgaagctaacgccctcttagagagcgtcactaagttccactgcggggttatctac                                                                                                                                                                                                                  <  1:272445‑M1/301‑70 (MQ=255)`  
                                                                           `ataagagacaggaagttaggccaactGCTGGTTCGTATGTACAACGTGGAAGCCAAGCGACTGAACGATGAGGCTCGTAAAGAGGCCACACAGTCACGCGCTCTGGCGATTCGCTCAAACGAACTGGCTGACAGTGCATCCACTAAAGTTACCGAGGCTGCCCGTGTGGCAAACCAAGCTCAACAGCTTTCCAAATTCTTTGAGTAATCAAACA‑GGAGAAACCATTATGatcgtttctgacatcgaagctaacgccctcttagagagcgtcactaagttccactgcggggttatctacgac                                                                                                                                                                                                               <  1:221248‑M1/290‑73 (MQ=255)`  
                                                                           `ataagagaaaggaagttaggccaactGCTGGTTCGTATGTACAACATGGAAGCCAAGCGACTGAACGATGAGGCTCGTAAAGAGGCCACACAGTCACGCGCTCTGGCGATTCGCTCCAACGAACTGGCTGACAGTGCATCCACTAAAATTACCGAGGCTGCCCGTGTGGCAAACCAAGCTCAACAGCTTTCCAAATTCTTTGAGTAATCAAACA‑GGAGAAACCATTATGatcgtttctgacatcgaagctaacgccctcttagagagcgtcactaagttccactgcggggttatctacgac                                                                                                                                                                                                               <  1:306238‑M1/290‑73 (MQ=255)`  
                                                                           `agacagattaaGAAGTTAGGCCCACAGCTGGTTCGTATGTACAACGTGGAAGCCAAGCGACTGAACGATGAGGCTCGTAAAGAGGCCACACAGTCACGCGCTCTGGCTATTCGCTCCAACGAACTGGCTGACAGTGCATCCACTAAAGTTACCGAGGCTGCCCGTGTGGCAAACCAAGCTCAACAGCTTTCCAAATTCTTTGAGTAATCAAACA‑GGAGAAACCATTATGatcgtttctgacatcgaagctaacgccctcttagagagcgtcactaagttccactgcggggttatctacgac                                                                                                                                                                                                               <  1:224834‑M1/296‑73 (MQ=255)`  
                                                                           `aaaCTGATTAAGAAGTTAGGCCAACTGCTGGTTCGTATGTACAACGTGGAAGCCAAGCGACTGAACGATTAGGCTCGTAAAGAGGCCACATAGTCACGCGCTCTGGCGATTCGCTCCAACGAACTGGCTGACAGTGCATCCACTAAAGTTACCGAGGCTGCCCGTGTGGCAAACCAAGCTCAACAGCTTTCCAAATTCTTTGAGTAATCAAACA‑GGAGAAACCATTATGatcgtttctgacatcgaagctaacgccctcttagagagcgtcactaagttccactgcggggttatctacgac                                                                                                                                                                                                               <  1:72539‑M1/301‑73 (MQ=255)`  
                                                                           `aaaCTGATTAAGAAGTTAGGCCAACTGCTGGTTCGTATGTACAACGTGGAAGCCAAGCGACTGAACGATGAGGCTCGTAAAGAGGCCACACAGTCACGCGCTCTGGCGATTCGCTCCAACGAACTGGCTGACAGTGCATCCACTAAAGTTACCGAGGCTGCCCGTGTGGCAAACCAAGCTCAACAGCTTTCCAAATTCTTTGAGTAATCAAACA‑GGAGAAACCATTATGatcgtttctgacatcgaagctaacgccctcttagagagcgtcactaagttccactgcggggttatctacgac                                                                                                                                                                                                               <  1:147220‑M1/301‑73 (MQ=255)`  
                                                                               `tgatgaAGAAGTTAGGCCAACTGCTGGTTCGTATGTACAACGTGGAAGCCAAGCGACTGAACGATGAGGCGCGTAAAGAGTCCACACAGTCACGCGCTCTGGCGATTCGCGCCAACGAACTGGCTGACAGTGCATCCACTAAAGTTACCGAGGCTGCCCGTGTGGCAAACCAAGCTCAACAGCTTTCCAAAGTCTTTGAGTAATCAAACA‑GGAGAAA‑CATTATGatcgtttctgacatcgaagctaactccctcttagagagcgtcactaagttccactgcggggttatctacgactactc                                                                                                                                                                                                          <  1:139594‑M1/301‑78 (MQ=255)`  
                                                                                   `tAAGAAGTTAGGCCAACTGCTGGTTCGTATGGACAACGTGGAAGCCAAGCGACTGAACGATGAGGCTCGTAAAGAGGCCACACAGTCACGCGCTCTGGCGATTCGCTCCAACGAACTGGCTGACAGTGCATCCACTAAAGTTACCGAGGCTGCCCGTGTGGCAAACCAAGCTCAACAGCTTTCCAAATTCTTTGAGTAATCAAACA‑GGAGAAACCATTATGatcgtttctgacatcgaagctaacgccctcttagagagcgtcactaagttccactgcggggttatctacgactactccac                                                                                                                                                                                                       <  1:435976‑M1/301‑81 (MQ=255)`  
                                                                                       `aagTTAGGCCAACTGCTGGTTCGTATGTACAACGTGGAAGCCAAGCGACTGAACGATGAGGCTCGTACAGAGGCCACACAGTCACACGCTCTGTCGATTCGCTCCAACGCACTGGCTGACAGTGCATCCACTAAAGTTACCGAGGCTGCCCGTGTGGCAAACCAAGCTCAACCGCTTTCCAAATTCTTTGAGTAATCAAACA‑GGAGAAACCATTCTGatcgtttctgacatctaaactaacgccctcttagagagcgtcactacattccactgcggggttatctacgactactccaccgct                                                                                                                                                                                                   >  1:464879‑M1/1‑217 (MQ=255)`  
                                                                                        `agTTAGGCCAACTGCTGGTTCGTATGTACAACGTGGAAGCCAAGCGACTGAACGATGAGGCTCGTAAAGAGGCCACACAGTCACGCGCTCTGGCGATTCGCTCCAACGAACTGGCTGACAGTGCATCCACTAAAGTTACCGAGGCTGCCCGTGTGGCAAACCAAGCTCAACAGCTTTCCAAATTCTTTGAGTAATCAAACA‑GGAGAAACCATTATGatcgtttctgacatcgaagctaactccctcttagagagcgtcactaagttccactgcggggtgatctacgactactccaccgcct                                                                                                                                                                                                  >  1:292966‑M1/1‑216 (MQ=255)`  
                                                                                               `ccAACTGCTGGTTCGTATGTACAACGTGGAAGCCAAGCGACTGAACGATGAGGCTCGTAAAGAGGCCACACAGTCACGCGCTCTGGCGATTCGCTCCAACGAACTGGCTGACAGTGCATCCACTAAAGTTACCGAGGCTGCCCGTGTGGCAAACCAAGCTCAACAGCTTTCCAAATTCTTTGAGTAATCAAACA‑GGAGAAATCAATATGatcgtttctgacatcgaagctaacgccctcttagagagcgtcactaagttccactgcggggttatctacgactactccaccgctgagtacgt                                                                                                                                                                                           >  1:392118‑M1/1‑209 (MQ=255)`  
                                                                                                  `aCTGCTGGTTCGTATGTACAACGTGGAAGCCAATCGACTGAACGATGAGGCTCGTAAAGAGGCCACACAGTCACGCGCTCTGGCGATTCGCTCCAACGAACTGGCTGACAGTGCATCCACTAAAGTTACCGAGGCTGCCCGTGTGGCAAACCAAGCTCAACAGCTTTCCAAATTCTTTGAGTAATCAAACA‑GGAGAAACCATTATGatcgtttctgacatcgaagctaacgccctcttagagagcgtcactaagttccactgcggggttatctacgactactccaccgctgagtacgtaag                                                                                                                                                                                        <  1:77707‑M1/301‑96 (MQ=255)`  
                                                                                                      `ctgGATCGAATGTACAACGGGGAAGCCAAGCGACTGAACGATGAGGCTCGTAAAGAGGCCACACAGTCACGCGCTCGGGCGATTCGATCCAACGAACTGGCTGACAGTGCATCCACTAAAGTTACCTAGGCTGCCCGTGTGGCAAACCAGGCGCAACTGCTTTCCAAATTCTTGGAGTAATCAAACA‑GGAGAAACCATTATGatcgtgtctgacatcgaagctaacgccctcttagagagcgtcactaagttccactgcggggttatctacgactactccaccgctgagtacgtaagctac                                                                                                                                                                                    <  1:27761‑M1/301‑100 (MQ=255)`  
                                                                                                              `tATTTACAACCTGGAAGCCAAGCGACTGAACGATGAGGCTCGTAAAGAGGCCACACAGTCACGCGCTCTGGCGAGTCGCTCCAACGAACTGGCTGACAGTGCATCCACTAAAGTTACCGAGGCTGCCCGTGTGGCAAACCAAGCTCAACAGCTTTCCAAATTCTTTGAGTAATCAAACA‑GGAGAAATCAATATGatcgtttctgacatcgaagctaacgccctcttagagagcgtcactaagttccactgcggggttatctacgactactcaacagctgagtacgtaagctaccgtccgag                                                                                                                                                                            <  1:19445‑M1/301‑108 (MQ=255)`  
                                                                                                                `tGTACAACGTGGAAGCCAAGCGACTGAACGATGAGGCTCGTAAAGAGGCCACACAGTCACGCGCTCTGGCGATTCGCTCCAACGAACTGGCTGACAGTGCATCCACTAAAGTTACCGAGGCTGCCCGTGTGGCAAACCAAGCTCAACAGCTTTCCAAATTCTTTGAGTAATCAAACA‑GGAGAAACCATTATGatcgtttctgacatcgaagctaacgccctcttagagagcgtcactaagttccactgcggggttatctacgactactccaccgctgagtacgtaagctacctgtctctta                                                                                                                                                                          >  1:263304‑M1/1‑192 (MQ=255)`  
                                                                                                                     `aaCGAGGAAGCCAAGCGACTGAACGATGAGGCTCGTAAAGAGGCCACACAGTAACGCGCTCTGGCGATTCGCTCCAACGAACTGGCTGACAGTGCATCCACTAAAGTTACAGAGGCTGCCCGTGTGGCAAACCAAGCTCAACAGCTTTCCAAATTCTTTGAGTAATCAAACA‑GGAGAAACAATTATGatcgtttctgacatcgaagctaacgccctcttagagagcgtcactaagttccactgcggggttatctacgactactccaccgctgagtacgtaagctaccgtccgagtgacttc                                                                                                                                                                     <  1:508719‑M1/301‑115 (MQ=255)`  
                                                                                                                        `gTGGAAGCCAAGCGACTGAACGATGAGGCTCGTAAAGAGGCCACACAGTCACGCGCTCTGGCGATTCGCTCCAACGAACTGGCTGACAGTGCATCCACTAAAGTTACCGAGGCTGCCCGTGTGGCAAACCAAGCTCAACAGCTTTCCAAATTCTTTGAGTAATCAAACA‑GGAGAAACCATTATGatcgtttctgacatcgaagctaacgccctcttagagagcgtcactaagttccactgcggggttatctacgactactccaccgctgagtacgtaagctaccgtccgagtgacttcggt                                                                                                                                                                  >  1:484921‑M1/1‑184 (MQ=255)`  
                                                                                                                               `ccAAGCGACTGAACGATGAGGCTCGTAAAGAGGCCACACAGTCACGCGCTCTGGCGATTCGCTCCAACGAACTGGCTGACAGTGCATCCACTAAAGTTACCGAGGCTGCCCGTGTGGCAAACCAAGCTCAACAGCTTTACAAATTCTTTGAGTAATCAAACA‑GGAGAAACCATTATGatcgtttctgacatcgaagctaacgccctcttagagagcgtcactaagttccactgcggggttatctacgactactccaccgctgagtacgtaagctaccgtccgagtgacttcggtgcgtatc                                                                                                                                                           <  1:427787‑M1/301‑125 (MQ=255)`  
                                                                                                                                   `gCGACTGAACGATGAGGCCCGTAAAGAGGCCACACAGTCACGCGCTCTGGCGATTCGCTCCAACGAACTGGCTGACAGTGCATCCACTAAAGTTACCGAGGCTGCCCGTGTGGCAAACCAAGCTCAACAGCTTTCCAAATTCTTTGAGTAATCAAACA‑GGAGAAACCATTATGatcgtttctgacatcgaagctaacgccctcttagagagcgtcactaagttccactgcggggttatctacgactactacaccgctgagtacgtaagctaccgtccgagtgacttcggtgcgtatctgga                                                                                                                                                       <  1:121570‑M1/301‑129 (MQ=255)`  
                                                                                                                                    `cGACTGAACGATGAGGCTCGTAAAGAGGCCACACAGTCACGCGCTCTGGCGATTCGCTCCAACGAACTGGCTGACAGTGCATCCACTAAAGTTACCGAGGCTGCCCGTGTGGCAAACCAAGCTCAACAGCTTTCCAAATTCTTTGAGTAATCAAACA‑GGAGAAACCATTATGatcgtttctgacatcgaagctaccgccctcttaaggcgcttcactaagttccactgcggggttatctacgactactccaccgctgagtccttactctaccgtccgattgccttcggtgcgtatcctttc                                                                                                                                                      >  1:310219‑M1/1‑172 (MQ=255)`  
                                                                                                                                     `gACTGAACGATGAGGCTCGTAAAGAGGCCACACAGTCACGCGCTCTGGCGATTCGCTCCAACGAACTGGCTGACAGTGCATCCACTAAAGTTACCGAGGCTGCCCGTGTGGCAAACCAAGCTCAACAGCTTTCCAAATTCTTTGAGTAATCAAACA‑GGAGAAACCATTATGatcgtttctgacatcgaagctaacgccctcttagagatcgtcactaagttccactgcggggttatctacgactactccaccgctgagtacgtaagctaccgtccgagtgacttcggtgcgtatctggatg                                                                                                                                                     <  1:156790‑M1/301‑131 (MQ=255)`  
                                                                                                                                         `gAACGATGAGGCTCGTAAAGAGGCCACACAGTCACGCGCTCTGGCGATTCGCTCCAACGAACTGGCTGACAGTGCATCCACTAAAGTTACCGAGGCTGCCCGTGTGGCAAACCAAGCTCAACAGCTTTCCAAATTCTTTGAGTAATCAAACA‑GGAGAAATCAATATGatcgtttctgacatcgaagctaacgccctcttagagagcgtcactaagttccactgcggggttatctacgactactccaccgctgagtacgtaagctaccgtccgagtgacttcggtgcgtatctggatgcgct                                                                                                                                                 >  1:198957‑M1/1‑167 (MQ=255)`  
                                                                                                                                         `gAACGATGAGGCTCGTAAAGAGGCCACACAGTCACGCGCTCTGGCGATTCGCTCCAACGAACTGGCTGACAGTGCATCCACTAAAGTTACCGAGGCTGCCCGTGTGGCAAACCAAGCTCAACAGCTTTCCAAATTCTTTGAGTAATCAAACA‑GGAGAAACCATTATGatcgtttctgacatcgaagctaacgccctcttagagagcgtcactaagttccactgcggggttatctacgactactccaccgctgagtacgtaagctaccgtccgagtgacctcggtgcgtatctggatgcgct                                                                                                                                                 >  1:337493‑M1/1‑167 (MQ=255)`  
                                                                                                                                            `cGATGAGGCTCGTAAAGAGGCCACACAGTCACGCGCTCTGGCGATTCGCTCCAACGAACTGGCTGACAGTGCATCCACTAAAGTTACCGAGGCTGCCCGTGTGGCAAACCAAGCTCAACAGCTTTCCAAATTCTTTGAGTAATCAAACA‑GGAGAAACCATTATGatcgtttctgacatcgaagctaacgccctcttagagagcgtcactaagttccactgcggggttatctacgactactccaccgctgagtacgtaagctaccgtccgagtgacttcggtgcgtatctggatgctctgga                                                                                                                                              >  1:461106‑M1/1‑164 (MQ=255)`  
                                                                                                                                             `gATGAGGCTCGTAAAGAGGCCACACAGTCACGCGCTCTGGCGATTCGCTCCAACGAACTGGCTGACAGTGCATCCACTAAAGTTACCGAGGCTGCCCGTGTGGCAAACCAAGCTAAACAGCTTTCCAAATTCTTTGAGTAATCAAACA‑GGAGAAACCATTATGatcgtttctgacatcgaagctaacgccctcttagagagcgtcactaagttccactgcggggttatctacgactactccaccgctgagtacgtaagctaccgtccgagtgacttcggtgcgtatctggatgcgctggaa                                                                                                                                             <  1:359145‑M1/301‑139 (MQ=255)`  
                                                                                                                                                   `aagagacaggaggccacacagTCACGAGCTCTGGCGATTCGCTCCAACGAACTGGCTGACAGTGCATCCACTAAAGTTACCGAGGCTGCCCGTGTGGCAAACCAAGCTCAACAGCTTTCCAAATTCTTTGAGTAATCAAACA‑GGAGAAACCATTATGatcgtttctgacatcgaagctaacgacctcttagagagcgtcactaagttccactgcggggttatctacgactactccaccgctgagtacgtaagctaccgtccgagtgacttcggtgcgtatctggatgcgctggaagccgag                                                                                                                                       <  1:248165‑M1/292‑145 (MQ=255)`  
                                                                                                                                                         `tgtataagagacagggcacgcgctctggcgattCGCTCCAACGAACTGGCTGACAGTGCATCCACTAAAGTTACCGAGGCTGCCCGTGTGGCAAACCAAGCTCAACAGCTTTCCAAATTCTTTGAGTAATCAAACA‑GGAGAAATCAATATGatcgtttctgacattgaagctaacgccctcttagagagcgtcactaagttccactgcggggttatctacgactactccaccgctgagtacgtaagctaccgtccgagtgacttcggtgcgtatctggatgcgctggaagccgaggttgca                                                                                                                                 <  1:332826‑M1/285‑151 (MQ=255)`  
                                                                                                                                                          `cagaggCCACACGGTCACGCGCTCTGGCTATTCGCTCCAACGAACTGGCTTACAGTGCATCCACTAAAGTTCCCGAGCCTGCCCGTGTTGCAAACCAAGCTCAACAGCTTTCCAAATTCGTTGAGTACTCAAACA‑GGAGAAACCATTATGatcgattctgacagcgaagctaacgccctcttagagagcgtcactaagttccactgcggggttatctacgactactccaccgctgagtacgtaagctaccgtccgagtgacttcggtgcgtatctggatgcgctggaagccgaggttgcac                                                                                                                                <  1:243963‑M1/300‑152 (MQ=255)`  
                                                                                                                                                              `ggCCACATAGTCACGCGCTCTGGCGATTCGCTCCAACGAACTGGCTGACAGTGCATCCACTAAAGTTACCGAGGCTGCCCGTGTGGCAAACCAAGCTCAACAGCTTTCCAAATTCTTTGAGTAATCAAACA‑GGAGAAACCATTATGatcgtttctgacatcgaagctaacgccctcttagagagcgtcactaagttccactgcggggttatctacgactactccaccgctgagtacgtaagctaccgtccgagtgacttcggtgcctgtctcttatacacatctccgagcccacgagaccc                                                                                                                            >  1:456099‑M1/1‑146 (MQ=255)`  
                                                                                                                                                                   `agcagtCACGCGCGCTGGCGATTCGCTCCAACGAACTGGCTGACAGTGCATCCACTAAAGTTACCGAGGCTGCCCGTGTGGCAAACCAAGCTCAACAGCTTTCCAAATTCTTTGAGTAATCAAACA‑GGAGAAATCAATATGatcgtttctgacatcgaagctaacgccctcttagagagcgtcactaagttccactgcggggttatctacgactactccaccgctgagtacgtaagctaccgtccgagtgacttcggtgcgtatctggatgcgctggaagccgaggttgcacgaggcggtc                                                                                                                       <  1:91271‑M1/299‑161 (MQ=255)`  
                                                                                                                                                                       `gTCACGCGCTCTGGCGATTCGCTCCAACGAACTGGCTGACAGTGCATCCACTAAAGTTACCGAGGCTGCCCGTGTGGCAAACCAAGCTCAACAGCTTTCCAAATTCTTTGAGTAATCAAACA‑GGAGAAACCATTATGatcgtttctgacatcgaagctaacgccctcttagagagcgtcactaagttccactgcggggttatctacgactactccaccgctgagtacgtaagctaccgtccgagtgacttcggtgcgtatctggatgcgctggaagccgaggttgcacgaggcggtcttat                                                                                                                   >  1:152016‑M1/1‑137 (MQ=255)`  
                                                                                                                                                                         `cACGCGCTCTGGCGATTCGCTCCAACGAACTGGCTGCCAGTGCATCCACTAAAGTTACCGAGGCTGCCCGTGTGGCAAACCAAGCTCAACAGCTTTCCAAATTCTTTGAGTAATCAAACA‑GTAGAAACCATTATGatcgtttctgacatcgaagctaacgccctcttagagagcgtcactaagttccactgcggggttatctacgactactccaccgctgagtacgtaagctaccgtccgagtgacttcggtgcgtatctggatgcgctggaagccgaggttgcacgaggcggtcttattg                                                                                                                 <  1:143203‑M1/301‑167 (MQ=255)`  
                                                                                                                                                                         `cACGCGCTCTGGCGATTCGCTCCAACGAACTGGCTGACAGTGCATCCACTAAAGTTACCGAGGCTGCCCGTGTGGCAAACCAAGCTCAACAGCTTTCCAAATTCTTTGAGTAATCAAACA‑GGAGAAACCATTATGatcgtttctgacatcgaagctaacgccctcttagagagcgtcactaagttccactgcggggttatctacgactactacaccgctgagtacgtaagctaccgtccgagtgacttcggtgcgtatctggatgcgctggaagccgaggttgcacgaggcggtcttattg                                                                                                                 <  1:215467‑M1/301‑167 (MQ=255)`  
                                                                                                                                                                         `cACGCGATCTGCGGATTCGCTCCAACGAACTGGCTGACAGTTCATCCACTAAAGTTACCGAGGCGGCCCGTGTGGCAAACCAAGCTCAACAGCTTTCCAAATTCTTTGAGTAATCAAACA‑GGAGAAACCATTATGatcgtttctgacatcgaagctaacgccctcttagagagcgtcactaagttccactgcggggttatctacgactactccaccgctgagtacgtaagctaccgtccgagtgacttcggtgcgtatctggatgcgctggaagccgaggttgcacgaggcggtcttattg                                                                                                                 <  1:184859‑M1/301‑167 (MQ=255)`  
                                                                                                                                                                         `agacatgtatgacgattcgcgctaaCGAACTGGCTGCAAGTGCAGTCACTAAAGTTACCGAGGCTGCCCGTGTGGCAAACCAAGCTCAACAGCTTTCCAAATTCTTCGAATAATCAAACA‑GGAGAATCCATTATGatcgtttcttacatcgaagctaacgccctcttcgagagcgtcaataagttccactgcggggttatctacgactactccaccgctgattacgtaagctaccgtccgagtgacttcggtgcgtatctggatgcgctggaagccgaggttgcacgaggcggtcttattg                                                                                                                 <  1:503804‑M1/289‑167 (MQ=255)`  
                                                                                                                                                                            `gcgcTCTGGCGATTCGCTCCAACGAACTGGCTGACAGTGCATCCACTAAAGTTACCGAGGCTGCCCGTGTGGCAAACCAAGCTCAACAGCTTTCCAAATTCTTTGAGTAATCAAACA‑GGAGAAACCATTATGatcgtttctgacatcgaagctaacgccctcttagagagcgtcactaagttccactgcggggttatctacgactactccaccgctgagtacgtaagctaccgtccgagtgacttcggtgcgtatctggatgcgctggaagccgaggttgcacgaggcggtcttattgtgt                                                                                                              <  1:351560‑M1/301‑170 (MQ=255)`  
                                                                                                                                                                             `aacactgacgATACGCTCCAACGAACGGGATGACAGTTCATCCACTAAAGTTACCGGGGCTACCCGTGTGGCAAACCAAGCACAACAGC‑TGCCAAATTCTTAGAGAAAACAAACA‑GGAGAAACAAGTATGaacgtgtcggatatcgaagctaacgccctattagagagaatcactaagtaccactgcggggttatctacaactactccaccgctgagtacgtaatctaccgtccgagtgacttcggtgcgtatctagatgcgcttgaagccgaggttgcacgaggcggtcttattgtgttc                                                                                                            <  1:281662‑M1/297‑172 (MQ=255)`  
                                                                                                                                                                                 `ctGGCGATTCGCTCCAACGAACTGGCTGACAGTGCATCCACTAAAGTTACCGAGGCTGCCCGTGTGGCAAACCAAGCTCAACAGCTTTCCAAATTCTTTGAGTAATCAAACA‑GGAGAAACCATTATGaacgtttctgacatcgaagctaacgccctcttagagagcgtcactaagttccactgcggggttatctacgactactccaccgctgagtacgtaagctaccgtccgagtgacttcggtgcgtatctggatgcgctggaagccgaggttgcacgaggcggtcttattgctgtctct                                                                                                         >  1:314764‑M1/1‑127 (MQ=255)`  
                                                                                                                                                                                   `ggCGATTCGCTCCAACGAACTGGCTGACAGTGCATCCACTAAAGTTACCGAGGCTGCCCGTGTGGCAAACCAAGCTCAACAGCTTTCCAAATTCTTTGAGTAATCAAACA‑GGAGAAATCAATATGatcgtttctggcctcgaagctaacgccctcttagagatcgtcactaagttccactgcggggttatctacgactactccaccgctgagtacgtaagctaccgtccgagtgacatcggtgcgtatccggatgcgctggaagccgagcttgaacgaggcctgcctcttatacccatctc                                                                                                       >  1:238056‑M1/1‑125 (MQ=255)`  
                                                                                                                                                                                             `gccaACGAACTGGCTGACAGTGCATCCACTAAAGTTACCGAGGCTGCCCGTGTGGCAAACCAAGCTCAACAGCTTTCCAAATTCTTTGAGTAATCAAACA‑GGAGAAACCATTATGatcgtttctgacatcgaagctaacgccctcttagagagcgtcactaagctccactgcggggttatctacgactactccaccgctgagtacgtaagctaccgtccgagtgacttcggtgcgtatctggatgcgctggaagccgaggttgcacgaggcggtcttattgtgttccacaacggtcacaag                                                                                             <  1:230792‑M1/300‑187 (MQ=255)`  
                                                                                                                                                                                             `cccaACGAACTGGCTGACAGTGCATCCCCTAAATGTACCGAGGCGGCCCGTGGGGAAAACCAAGCTCAACAGCTTTCCAAAGTCTTTGAGTAATCAAAAC‑GGGGAAACCATTATGatcgtttcggacatcgaagctaacgccctcttagagcgcgtcactaagttccactgcggggttatctacgactactccaccgctgagtacgtaagctaccgtccgagtgacttcggtgcgtatctggatgcgctggaagccgaggttgcacgaggcggtcttattgtgttccacaacggtcacaag                                                                                             <  1:398808‑M1/300‑187 (MQ=255)`  
                                                                                                                                                                                              `ccAACGAACTGGCTGACAGTGCATCCACTAAAGTTACCGAGGCTGCCCGTGTGGCAAACCAAGCTCAACAGCTTTCCAAATTCTTTGAGTAATCAAACA‑GGAGAAACCATTATGatcgtttctgacatcgaagctaacgccctcttagagagcgtcactaagttccactgcggggttatctacgactactccaccgctgagtacgtaagctaccgtccgagtgacttcggttcgtatctggatgcgctggaagccgaggttgcacgaggcggtcttattgtgttccacaacggctttctct                                                                                            >  1:52415‑M1/1‑114 (MQ=255)`  
                                                                                                                                                                                                  `cGAACTGTCTGACAGTTCATCCACTAAAGTTACCGAGGCTGCCCGTTTGTCAAACCAAGCTCAACAGCTTTCCAAATTCTTTGAGTAATCAAACA‑GGAGAAACCATTATGatcgtttctgacatcgaatctaacgccatcttagagagcgtcactaagttccactgcggggttatctacgactactccaccgctgagtacgtaagctaccgtccgagtgacttcggtgcgtatctggatgcgctggaagccgaggttgcacgaggcggtcttattgtgttccacaacggtcacaagtatga                                                                                        <  1:255849‑M1/301‑192 (MQ=255)`  
                                                                                                                                                                                                   `aaacTGGCTGACAGTGCATCCACTAAAGTTACCGAGGCTGCCCGTGTGGCAAACCAAGCTCAACAGCTTTCCAAATACTTGGAGTAATCAAACA‑GGAGAAAGCAATATGctcgtttttgacatcgaagctaacgccctcttagatagcgtcactaagtcccactgcggggttatctacgactactccaccgctgagtacgtaatctaccgtccgagtgacttcgatgcgtatctggatgcgctggaagccgaggttgcacgaggcggtcttattgtgttccacaacggtcacaagtatgac                                                                                       <  1:95322‑M1/300‑193 (MQ=255)`  
                                                                                                                                                                                                     `agagacagacagtgcATCCACTAAAGTTACCGAGGCTGCACGTGTGGCAAACCAAGCTCAACAGCTTTACAAATTCTTTGAGTAATCAAACA‑GGAGAAACCATTATGatcgtttctgacatcgaagctaacgccctcttagagggcgtcactaagttccactgcggggttatctacgactactccaccgctgagtacgtaagctaccgtccgagtgacttcggtgcgtatctggatgcgctggaagccgaggttgcacgaggcggtcttattgtgttccacaacggtcacaagtatgacgt                                                                                     <  1:376781‑M1/294‑195 (MQ=255)`  
                                                                                                                                                                                                      `cTGGCTGACAGTGCATCCACTAAAGTTACCGAGGCTGCCCGTGTGGCAAACCAAGCTCAACAGCTTTCCAAATTCTTTGAGTAATCAAACA‑GGAGAAACCATTATGatcgtttctgacatcgaagctaacgccctcttagagagcgtcactaagttccactgcggggttatctacgactactccaccgctgagtacgtaagctaccgtccgagtgacttcggtgcgtatctggatgcgctggaagccgaggtttcacgaggcggtcttattgtgttccacaacggtcacaagtatgacgtt                                                                                    >  1:133797‑M1/1‑106 (MQ=255)`  
                                                                                                                                                                                                        `ggCTGACAGTGCATCCACTAAAGTTACCGAGGCTGCCCGTGTGGCAAACCAAGCTCAACAGCTTTCCAAATTCTTTGAGTAATCAAACA‑GGAGAAACCATTATGatcgtttctgacatcgaagctaacgccctcttagagagcgtcactaagttccactgcggggttatctacgactactccaccgctgagtacgtaagctaccgtccgagtgacttcggtgcgtatctggatgcgctggaagccgaggttgcacgaggcggtcttattgtgttccacaacggtcacaaggataaatgtcc                                                                                  >  1:40455‑M1/1‑104 (MQ=255)`  
                                                                                                                                                                                                          `cagacAGGGCATCCACTAAAGTTACCGAGGCTGCCCGTGTGGCAAACCAAGCTCAACAGCTGTCCAAATTCTTTGAGTAATCAAACA‑GGAGAAACCATTATGatcgtttctgacatcgaagctaacgccctcttagagagcgtcactaagttccactgcggggttatctacgactactcaaccgctgagtacgtaagctaccgtccgagtgacttcggtgcgtatctggatgcgctggaagccgaggttgcacgaggcggtcttattttgttccacaacggtcacaagtatgacgttcctg                                                                                <  1:303025‑M1/299‑200 (MQ=255)`  
                                                                                                                                                                                                             `caggtgcATCCACTAAAGTTACCGAGGCTGCCCGTGTGGCAAACCAAGCTCAACAGCTTTCCAAATTCTTTGAGTAATCAAACA‑GGAGAAACCATTATGatcgtttctgacatcgaagttaacgccctcttagagagcgtcactaagttccactgctgggttatctacgactactccaccgctgagtacgtaagctaccgtccgagtgacttcggtgcgtatctggatgcgctggaagccgaggttgcacgagtcggtcttattgtgttccacaacggtcacaagtatgacgttcctgcat                                                                             <  1:190587‑M1/298‑203 (MQ=255)`  
                                                                                                                                                                                                             `accggtcgtccactaaaGTTACCCAGGCTGCCCGTGTGGCAATCCAAGATCAACAGCTTTCAAAATTCTTTGAGTAATCAAACA‑GGAGAAACCATTATGatcgtttctgacatcgaagctaacgcccgcttagagagcgtcactaagttccactgcgtggttatctacgactactccccccctgagtacgtaagctaccgtccgagtgacttcggtgcggatctggatgcgctggaagccgaggttgcacgaggcggtcttattgtgttccacaacggttacaagtatgacgttcctgcat                                                                             <  1:346616‑M1/293‑203 (MQ=255)`  
                                                                                                                                                                                                             `aCAGTGCATCCACTAAAGTTACCGAGGCTGCCCGTGTGGCAAACCAAGCTCAACAGCTTTCCAAATTCTTTGAGTAATCAAACA‑GGAGAAACCATTATGatcgtttctgacatcgaagctaacgccctcttagagagcgtcactaagttccactgcggggttatctacgactactccaccgctgagtacgtaagctaccgtccgagtgacttcggtgcgtatctggatgcgctggaagccgaggttgcacgaggcggtcttattgtgttccacaacggtcacaagtatgacgttcctgcat                                                                             <  1:67028‑M1/301‑203 (MQ=255)`  
                                                                                                                                                                                                              `cAGTGCATCCACTAAAGTTACCGAGGCTGCCCGTGTGGCAAACCAAGCTCAACAGCTTTCCAAATTCTTTGAGTAATCAAACA‑GGAGAAACCATTATGatcgtttctgacatcgaagctaacgccatcttagagagcgtcactaagttccactgcggggttatctacgactactccaccgctgagtacgtaagctaccgtccgagtgacttcggtgcgtatctggatgcgctggaagccgaggttgcacgaggcggtcttattgtgttccacaacggtaacaagtatgacgttcctgaatt                                                                            >  1:363409‑M1/1‑98 (MQ=255)`  
                                                                                                                                                                                                              `cAGTGCATCCACTAAAGTTACCGAGGCTGCCCGTGTGACAAACCAAGCTCAACAGCTTTCCAAATTCTTTGAGTAATCAAACA‑GGAGAAACCATTATGatcgtttctgacatcgcagctaacgccctcttagagagcggcactaagttccactgcggggttatctacgactactccaccgctgagtacgtaagctaccgtccgagtgacttcggtgcgtatctggatgcgctggaagccgcggttgcacgaggccgtcttattgttttccccaacggtcgcaagtatgacgttccttcatt                                                                            >  1:101400‑M1/1‑98 (MQ=255)`  
                                                                                                                                                                                                                `gTGCATCCACTAAAGTTACCGAGGCTGCCCGTGTGGCAAACCAAGCTCAACAGCTTTCCAAATTCTTTGAGTAATAAAACA‑GGAGAAACCATTATGatcgtttctgacatcgaaactaacgccctcttagagagcgtcactaagttccactgcggggttatctacgactactccaccgctgagtacgtcagctaccgtccgagtgacttaggtgcgtatctggatgcgctggcagccgaggttgcacgaggcggtcttattgtgttccacaacggtcacaagtatgacgttcctgcattgc                                                                          >  1:372549‑M1/1‑96 (MQ=255)`  
                                                                                                                                                                                                                  `gCATCCACTAAAGTTACCGAGGCTGCCCGTGTGGCAAACCAAGCTCAACAGCTTTCCAAATTCTTTGAGTAATCAAACA‑GGAGAAATCAATATGatcgtttctgacatcgaagctaacgcccgcttagagagcgtcactaagttccactgcggggttatctacgactactccaccgctgagtacgtaagctaccgtacgagtgacttcggtgcgtatctggatgcgctggaagccgaggttgcatgaggcggtcttattgtgttccacaacggtcacaagtatgacgttcctgcattgacc                                                                        <  1:130625‑M1/301‑208 (MQ=255)`  
                                                                                                                                                                                                                         `cTAAAGTTACCGAGGCTGCCCGTGTGGCAAACCAAGCTCAACAGCTTTCCAAATTCTTTGAGTAATCAAACA‑GGAGAAATCAATATGatcgtttctgacatcgaagctaacgccctcttagagagcgtcactaagttccactgcggggttatctacgactactccaccgctgagtacgtaagctaccgtccgagtgacttcggtgcgtatctggatgcgctggaagccgaggttgcacgaggcggtcttattgtgttccacaacggtcacaagtatgacgttcctgcattgaccaaactgg                                                                 >  1:440285‑M1/1‑87 (MQ=255)`  
                                                                                                                                                                                                                         `cTAAAGTTACCGAGGCTGCCCGTGTGGCAAACCAAGCTCAACAGCTTTCCAAATTCTTTGAGTAATCAAACA‑GGAGAAACCATTATGatcgtttctgacatcgaagctaacgccctcttagagagcgtcactaagttccactgcggggttatctacgactactccaccgctgaggacgtaagctaccgtccgagtgacttcggtgcgtctctggatgagctggaagccgaggttgcacgaggcggtcttattgtgatccacaacggtcccaagtatgacgttcctgcactgtctcttataa                                                                 >  1:259892‑M1/1‑87 (MQ=255)`  
                                                                                                                                                                                                                              `gTTACCGAGGCTGCCCGTGTGGCAAACCAAGCTCAACAGCTTTCCAAATTCTTTGAGTAATC‑AATA‑GGAGAAATCAATATGatagtttctgacatcgaagctaacgccctcttagagagcgtcactaagttccactgcggggttatctacgactactccaccgctgagtacgtaagctaccgtccgagtgacttcggtgcgtatctggatgcgctggaagccgaggttgcacgaggcggtcttattgtgttccacaacggtcacaagtatgacgtctgtctcttatcaccatctcctcacc                                                           >  1:116503‑M1/1‑81 (MQ=255)`  
                                                                                                                                                                                                                                `tACCGAGGCTGCCCGTGTGGCAAACCAAGCTCAACAGCTTTCCAAATTCTTTGAGTAATCAAACA‑GGAGAAACCATTATGctcgtttctgacatcgaagctaacgccctcttagagagcgtcactaagttccactgcggggttatctacgactactccaccgctgagtccgtacgctaccgtccgagtgacttcggtgcgtatctggatgctctggaagccgaggttgcccgaggcggtcttattgtgttccacaccggtcacacgtattacgttcctgcattttccaaactggcacattt                                                          >  1:419133‑M1/1‑80 (MQ=255)`  
                                                                                                                                                                                                                                        `cTGCCCGTGTGGCAAACCAAGCTCAACAGCTTTCCAAATTCTTTGAGTAATCAAACA‑GGAGAAACCATTATGatcgtttctgacatcgaagctaacgccctcttagagagcgtcactaagttccactgcggggttatctacgactactccaccgctgagtacgtaagctaccgtccgagtgacctcggtgcgtatctggatgcgctggaagccgaggttgcacgaggcggtcttattgtgttccacaacggtcacaagtatgacgttcctgcattgaccaaactggcaaagttgcaattga                                                  >  1:320796‑M1/1‑72 (MQ=255)`  
                                                                                                                                                                                                                                          `gCCCGTGTGGCAAACCAAGCTCAACAGCTTTCCAAATTCTTTGAGTAATCAAACA‑GGAGAAATCAATATGatcgtttctgacatcgaagctaacgacctcttagagagcgtcactaagttccactgcggggttatctacgactactccaccgctgagtacgtaagctaccgtccgagtgacttcggtgcgtatctggatgcgctggaagccgaggttgcacgaggcggtcttattgtgttccacaacggtcacaagtatgacgttcctgcattgaccaaactggcaaagttgcaattgaac                                                >  1:443892‑M1/1‑70 (MQ=255)`  
                                                                                                                                                                                                                                                `gtgGCAAACCAAGCTCAACAGCTTTCCAAATTCTTTGAGTAATCAAACA‑GGAGAAACCATTATTatcgtttctgacatcgaagctaacgccctcttagagagcgtcactaagttccactgcggggttatctacgactactccaccgctgagtacgtaagctaccgtccgagtgacttcggtgcgtatctggatgcgctggaagccgaggttgcacgaggcggtcttattgtgttccacaacggtcacaagtatgacgttcctgcattgaccaaactggcaaagctgtctcttatacacatc                                          >  1:432013‑M1/1‑64 (MQ=255)`  
                                                                                                                                                                                                                                                        `ccAAGCTCAACAGCTTTCCAAATTCTTTGAGTAATCAAACA‑GGAGAAACCATTATGctcgtttctgacatcgaagctaacgccctcttagagagcgtcactaagttccactgcggggttatctacgactactccaccgctgagtacgtaagctaccgtccgagtgacttcggtgcgtatctggatgcgctggaagccgaggttgcacgaggcggtcttattgtgttccacaacggtcacaagtatgacgttcctgcattgaccaaactggcaaagttgcaattgaacagagagttccacct                                  <  1:179487‑M1/301‑246 (MQ=255)`  
                                                                                                                                                                                                                                                          `aaGCTCAACAGCTTTCCAAATTCTTTTAGTAATCAAACA‑GGAGAAATCAATATGatcgtttctgacatcgaagctaacgccctcttagagagcgtcactaagttccactgcggggtgatctacgactactccaccgatgagtacgtaagctaccgtccgagtgacttcggtgcgtatctggatgcgctggaagccgaggttgcacgaggcggtcttattgtgttccacaacggtcacaagtatgacgttcctgcattgaccaaactgacaaagttgcaattgaaccgagagttccaccttc                                <  1:492335‑M1/301‑248 (MQ=255)`  
                                                                                                                                                                                                                                                                               `tcggagatgtgtataagagacagaaaccattatgatcgtttctgacatcgaagctaacgccctcttagagagcgtcactaagttccactgcggggttatctacgactactccaccgctgagtacgtaagctaccgtccgagtgacttcggtgcgtatctggatgcgccggaagccgaggttgcacgaggcggtcttattgtgttccacaacggtcacaagtatgacgttcctgcattgaccaaactggcaaagttgcaattgaaccgagagttccaccttcctcgtgagaactgtattgac            <  1:473002‑M1/280‑268 (MQ=255)`  
                                                                                                                                                                                                                                                                                `gagacagtagtcaaacA‑GGAGAAACCATTATGatcgtttctgacatcgaagctaacgccctcttagagagcgtcactaagttccactgcggggttatctacgactactccaacgctgagtacgtaagctaccgtccgagtgacttcggtgcgtatctggatgcgctgggagccgaggttgcacgaggcggtcttattgtgttccacaacggtcacaagtatgacgttcctgcattgaccaaactggcaaagttgcaattgaaccgagagttccaccttcctcgtgagaactgtattgacac          <  1:355213‑M1/296‑270 (MQ=255)`  
                                                                                                                                                                                                                                                                                        `atgtgtataagagacagccattatgatcgtttctgacatcgaagctaacgccctcttagagagcgtcactaagttccactgcggggttatctacgactactccaccgctgagtacgtaagctaccgtccgagtgacttcggtgcgtatctggatgcgctggaagccgaggttgcacgaggcggtcttattgtgttccacaacggtcacaagtatgacgttcctgcattgaccaaactggcaaagttgcaattgaaccgagagttccaccttcctcgtgagaactgtattgacacccttgtg   <  1:395562‑M1/284‑277 (MQ=255)`  
                                                                                                                                                                                                                                                                                        `aaTCAAACA‑GGAGAAATCAATAGGatcgtttctgacattgaagctaacgccctcttagagagcgtcattaagttccactgcggggttatctacgactacgccaccgctgagtacgtaagctaccgtccgagtgacttcggtgcgtatctggatgcgctggaagccgaggttgcacgatgcggtcttattgtgttccacaacggtcacaagtatgacgttcctgcattgaccaaactggcaaagttgcaattgaaccgagagttccaccttcctcgtgagaactgtattgacacccttgtgt  <  1:106238‑M1/301‑278 (MQ=255)`  

                                                                                                                                                                                                                                                                                                            `|`                                                                                                                                                                                                                                                                                           
`TTCCAGACGACTACGAGAAGTTTCGCTGGAGAGTCCCATTCTAATACGACTCACTAAAGGAGACACACCATGTTCAAACTGATTAAGAAGTTAGGCCAACTGCTGGTTCGTATGTACAACGTGGAAGCCAAGCGACTGAACGATGAGGCTCGTAAAGAGGCCACACAGTCACGCGCTCTGGCGATTCGCTCCAACGAACTGGCTGACAGTGCATCCACTAAAGTTACCGAGGCTGCCCGTGTGGCAAACCAAGCTCAACAGCTTTCCAAATTCTTTGAGTAATCAAACA‑GGAGAAACCATTATGTCTAACGTAGCTGAAACTATCCGTCTATCCGATACAGCTGACCAGTGGAACCGTCGAGTCCACATCAACGTTCGCAACGGTAAGGCGACTATGGTTTACCGCTGGAAGGACTCTAAGTCCTCTAAGAATCACACTCAGCGTATGACGTTGACAGATGAGCAAGCACTGCGTCTGGTCAATGCGCTTACCAAAGCTGCCGTGACAGCAATTCATGAAGCTGGTCGCGTCAATGAAGCTATGGCTATCCTCGACAAGATTGATAACTAAGAGTGGTATC  >  NC_001604.1/13283‑13863`

  

Alignment Legend

Aligned base mismatch/match (shaded by quality score): `ATCG/ATCG` `< 3 ≤` `ATCG/ATCG` `< 9 ≤` `ATCG/ATCG` `< 19 ≤` `ATCG/ATCG` `< 33 ≤` `ATCG/ATCG` `< 38 ≤` `ATCG/ATCG`

Unaligned base: `atcg`    Masked matching base: `atcg`    Alignment gap: `‑`    Deleted base: `‑`
